# Integrated treadmill stress testing and MR relaxometry (T1, T2, T2*): response in healthy calf muscles

**DOI:** 10.1186/1532-429X-16-S1-P68

**Published:** 2014-01-16

**Authors:** Juliet Varghese, Debbie Scandling, Jason Craft, Subha V Raman, Orlando P Simonetti, Georgeta Mihai

**Affiliations:** 1 Dorothy M Davis Heart and Lung Research Institute, Wexner Medical Center, The Ohio State University, Columbus, Ohio, USA; 2 Department of Biomedical Engineering, The Ohio State University, Columbus, Ohio, USA; 3 Division of Cardiovascular Medicine, Department of Internal Medicine, Wexner Medical Center, The Ohio State University, Columbus, Ohio, USA; 4 Department of Radiology, Wexner Medical Center, The Ohio State University, Columbus, Ohio, USA

## Background

Peripheral arterial disease (PAD) is accompanied by a complex lower limb pathophysiology resulting in reduced functional capacity and quality of life. We aim to characterize the exercise recovery kinetics in the calf muscle of healthy volunteers by combining treadmill exercise and magnetic resonance (MR) relaxometry (T1, T2 and T2*), and to investigate their relation with age and treadmill exercise duration.

## Methods

**Figure 1:**
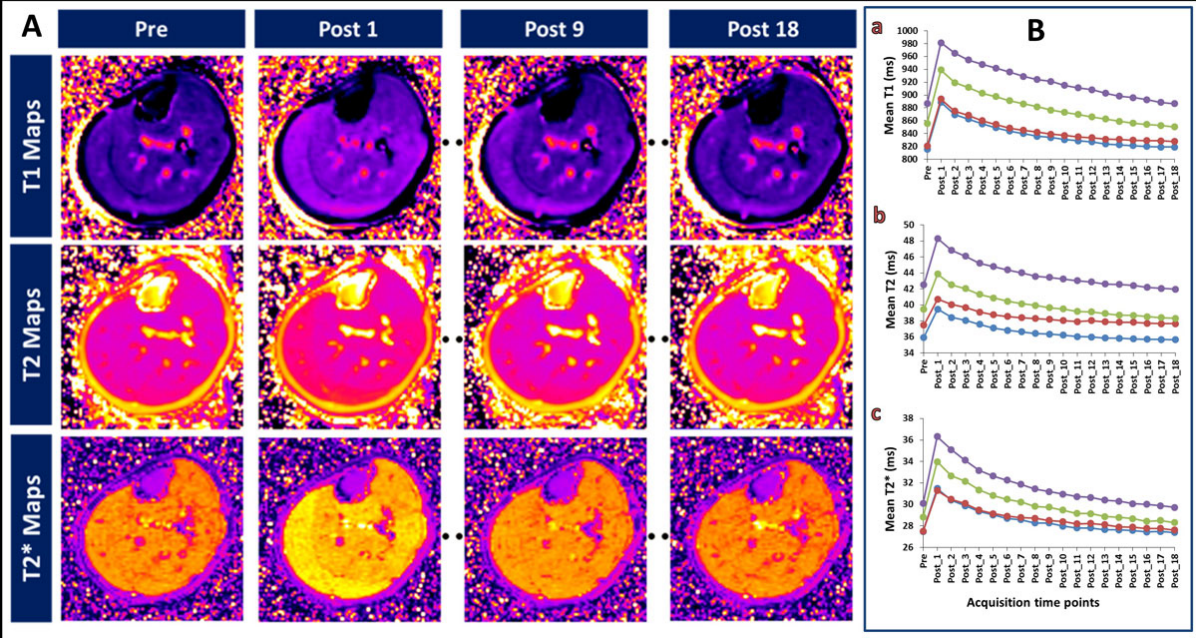
A. Time course of exercise induced changes in the left calf muscles of a 30 year old male volunteer. Axial T1 (top row), T2 (middle row) and T2* (bottom row) maps acquired before exercise (Pre), immediately after exercise (Post 1), at ~20 minutes (Post 9) and at ~45 minutes (Post 18) are shown. B. Exercise-induced time course changes of mean T1 (a), mean T2 (b) and mean T2* values (c) of the four calf muscle groups in 24 volunteers before (Pre) and after exercise (Post_1 to 18 - ~2:20 minute intervals). Graph in blue - TA, red - Sol, green - MG and purple - LG.

Twenty four healthy volunteers (age: 23-79 years, 12 males) performed Bruce treadmill stress test to at least 81% of age-predicted maximum heart rate on an MR-compatible treadmill adjacent to a 1.5T MRI system. Single slice axial T1 (TR/TE/TI = 1200/1.1/125, 205, 285, 1325, 1405, 1485, 2525, 2605, 2685, 3885 ms, FA = 35°, 1 NEX, 1.9 × 1.9 × 8 mm^3^, 1:02 min:s), T2 (TR/TE_T2p _= 2500/0, 24, 36, 48, 60 ms, FA = 70°, 2 NEX, 1.9 × 1.9 × 8 mm^3^, 50 s) and T2* (TR/TEs = 740/2.1, 4.2, 6.6, 8.9, 11.3, 13.7, 16.1, 18.4 ms, 2 NEX, FA = 18°, 1.9 × 1.9 × 8 mm^3^, 31 s) maps were sequentially acquired for ~15 minutes at rest, and for ~45 minutes post exercise (Figure 1A). The relaxometric values from small regions in anterior tibialis (TA), soleus (Sol), medial (MG) and lateral gastrocnemius (LG) muscles were recorded, averaged over both legs and plotted against time (Figure 1B). Rest, peak exercise, peak-rest difference, end of exercise recovery and decay rate values was checked against age and exercise test duration using Pearson's correlation.

## Results

**Figure 2:**
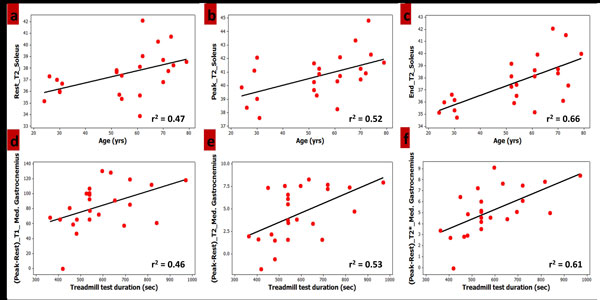
**Examples of significant correlations (p < 0.05) observed in relaxometric measures with age (a-c) and treadmill test duration (d-f)**.

T1, T2 and T2* demonstrated a significant increase from rest with exercise, followed by gradual recovery. T2 at rest (r^2^_TA = 0.48, r^2^_Sol = 0.47, r^2^_MG = 0.44, r^2^_LG = 0.53), peak T1, T2 and T2* (r^2 ^= 0.49, 0.52, and -0.52) in Sol, end of exercise T2 in Sol, MG and LG (r^2 ^= 0.66, 0.47, and 0.57) and T2 decay rate for TA (r^2 ^= 0.47) were related to age. The peak-rest differences of T1, T2 and T2* in MG (r^2 ^= 0.46, 0.53, and 0.61) and LG (r^2 ^= 0.58, 0.50, and 0.47) were correlated with test duration (Figure 2).

## Conclusions

We investigated treadmill exercise induced changes in normal calf muscles, and showed the exercise induced response can be quantified by MR relaxometry, and is age and exercise duration dependent. In future patient studies, we will explore the integration of the clinical indices of disease severity (ABI-Ankle Brachial Index, Initial and Absolute Claudication Distance) with MR relaxometric measures to enhance our understanding of the pathophysiology and severity of PAD.

## Funding

13PRE16950001.

